# Impact of the OncotypeDX score and HER2 RNA PCR levels on HER2-low IHC levels in primary and metastasized tumors

**DOI:** 10.1186/s12885-023-11530-w

**Published:** 2023-10-24

**Authors:** Didi Feldman, Liat Anabel Sinberger, Mali Salmon-Divon, Judith Ben-Dror, Shlomit Strulov Shachar, Amir Sonnenblick

**Affiliations:** 1 Faculty of Medicine, The Technion Institute of Technology, Haifa, Israel; 2 Department of Molecular Biology, Ariel University, Ariel, Israel; 3 Adelson School of Medicine, Ariel University, Ariel, Israel; 4 Division of Oncology, Tel Aviv Sourasky Medical Center, Tel Aviv, Israel; 5 School of Medicine, Tel Aviv University, Tel Aviv, Israel

**Keywords:** Breast cancer, HER2 low, Oncotype, HER2 negative, RT-PCR

## Abstract

**Purpose:**

One-half of hormone receptor-positive (HR +) breast cancer (BC) patients have low expression of HER2 (HER2-low) and may benefit from trastuzumab deruxtecan (TDXd). This study aimed to identify parameters associated with HER2-low levels in primary and metastatic tumors. We specifically sought to determine whether OncotypeDX and HER2 mRNA levels could identify patients who would otherwise be considered HER2-negative by immunohistochemistry (IHC).

**Methods:**

This retrospective analysis of all consecutive HR + patients who underwent OncotypeDX from January 2004 to December 2020 was conducted in a single medical center (*n* = 1429). We divided HER2-negative cases into HER2-low (IHC = 1 + or 2 + and non-amplified fluorescent situ hybridization) and HER2-0 (IHC = 0). HER2 RT-PCR was evaluated from the OncotypeDX results.

**Results:**

HER2-low cases exhibited significantly higher HER2 RT-PCR scores (*p* = 2.1e-9), elevated estrogen receptor (ER) levels (*p* = 0.0114), and larger tumor sizes compared to HER2-0 cases (> 2 cm; 36.6% vs. 22.1%, respectively, *p* < 0.00001). Primary tumors > 2 cm were more likely to be HER2-low (OR = 2.07, 95% CI: 1.6317 to 2.6475, *p* < 0.0001). Metastatic BCs expressed higher HER2 IHC scores compared with primary BCs (Wilcoxon signed-rank, *p* = 0.046). HER2 IHC scores were higher for low-risk vs. medium-risk OncotypeDX (*p* = 0.0067). No other clinical or pathological parameters were associated with the increase in HER2 levels in the metastatic samples.

**Conclusion:**

It might be beneficial to use clinical data from the primary tumor, including the HER2 RT-PCR score, to determine a HER2-low status.

## Introduction

Breast cancer (BC) is the most prevalent diagnosed malignancy worldwide [1]. It is a highly heterogeneous disease consisting of biological entities that differ in clinicopathologic features, susceptibility to treatments, and prognosis. Clinicians manage their decision-making approach based upon 3 main subtypes derived from immunohistochemistry (IHC) and fluorescent situ hybridization (FISH) analyses: luminal or HR-positive/HER2-negative (hormone receptor-positive/human epidermal growth factor receptor 2-negative), HER2-enriched and triple-negative or HR-negative/HER2-negative [2]. Overexpression of the HER2 subtype occurs in approximately 25% of BC cases [3]. HER2 is primarily divided into HER2-positive (IHC = 3 + or 2 + and FISH amplified) and HER2-negative (IHC = 0/1 + or 2 + and FISH not amplified). Targeted anti-HER2 therapies enabled an improvement in the prognosis of HER2-positive BC patients [4]. This dichotomic classification, however, benefited only a minority of BC patients, specifically, the HER2-positive subgroup [5]. Most HER2-negative BCs still express HER2 on cancer cells to some extent [6], differentiated HER2-low (IHC = 1 + or 2 + and FISH not amplified), which is now targetable, from HER2-0 (IHC = 0) [7].

Novel anti-HER2 agents for HER2-low BC, such as trastuzumab deruxtecan (TDXd; Enhertu, Daiichi Sankyo, Inc.), could improve prognosis for some patients currently excluded from HER2-targeted therapy, thereby expanding the reach to a much larger share of BC patients [7]. Patients diagnosed with HR + /HER2-"negative" early-stage BC undergo the OncotypeDX test to determine which patients will benefit from chemotherapy and to what degree. The OncotypeDX test quantifies the expression of 21 genes by means of real-time reverse transcription-polymerase chain reaction (qRT-PCR). One of them is the HER2 gene [8].

Despite its wide use by pathologists, the IHC test has some limitations. One factor that contributes to the discrepancy between labs is poor adherence to 2018 HER2 testing recommendations and faulty technical execution of the test. Another limitation might stem from insufficient sensitivity to accurately detect low levels of HER2 expression [6]. In the past, distinguishing between HER2 IHC 0 and IHC 1 + scores was not a primary concern for pathologists due to limited clinical impact. The introduction of targeted therapies such as TDXd has shifted this perspective, emphasizing the clinical significance of this differentiation. Accurate classification of HER2-low status now plays a vital role in guiding treatment decisions for optimal patient care. Consequently, there may be a need for careful reassessment of previous pathological reports.

All of these restraints might undermine the reliability of the test and consequently reduce the sensitivity of the HER2-low classification. Here, this study aimed to identify parameters that are associated with HER2-low levels in primary and relapsed tumors. We specifically sought to determine whether the RT-PCR test that measures HER2-mRNA expression could identify patients who could be classified as HER2-low.

## Materials and methods

### Study design, patient population, and data sources

The data for this single-center retrospective study were retrieved from the institutional OncotypeDX registry. They included the clinical and pathological features from the electronic medical records (EMR) of the Tel Aviv Sourasky Medical Center for patients diagnosed during January 2004–December 2020. Clinical and pathological data, as well as OncotypeDX score, were available from the same years. Excluded were HER2-positive patients. Data on the primary tumor and metastatic status (when available) included the patient's disease stage at diagnosis, age at diagnosis, and the following biological characteristics: tumor size, nodal status, ER expression (IHC), progesterone receptor expression (IHC), histological type, grade, OncotypeDX score, HER2 RT-PCR score, and whether the primary tumor had metastasized. For relapsed patients, we collected additional data on the use of adjuvant/neoadjuvant chemotherapy (CT) and CT regimen, the use, duration, and type of endocrine therapy (ET), the use of adjuvant radiotherapy (RT), location of the metastasis, time to relapse, menopausal status, and death (Tables 1 and 2).

**Table 1:** Patient and tumor characteristics

Charateristics	HER2-0 (*n* = 588)	HER2-low (1/2) (*n* = 841)	p^*^
Age at testing, years, median (IQR)	59 (49.75–65)	60(50–66)	0.64
Tumor size, cm, median (IQR)	1.5(1.2–2)	1.6(1.2–2.2)	**0.003**
Tumor size category, n			** < 0.00001**
≤ 1 cm	111(18.9%)	135(16%)	
> 1–2 cm	325(55.3%)	362(43%)	
> 2 cm	130(22.1%)	308(36.6%)	
Not available	22(3.7%)	36(4.3%)	
Tumor grade, n			0.327
Grade 1	44(7.4%)	57(6.8%)	
Grade 2	324(55.1%)	459(54.6%)	
Grade 3	110(18.7%)	188(22.3%)	
Not available	110(18.7%)	137(16.3%)	
Histology, n			0.227
IDC	464(78.9%)	677(80.5%)	
ILC	89(15.1%)	99(11.8%)	
Mucinous/colloid/papillary	16(2.7%)	23(2.7%)	
Other/not available	19(3.2%)	42(5%)	
Nodal status, n			0.223
N0	407(69.2%)	585(69.6%)	
N1mi	45(7.6%)	67(8%)	
N1	75(12.7%)	102(12.1%)	
N2	23(3.9%)	40(4.8%)	
N3	14(2.4%)	19(2.3%)	
N4 <	8(1.4%)	2(0.2%)	
Not available	16(2.7%)	26(3%)	
OncotypeDX median [IQR]	17[12–23]	17[12–23]	0.622
OncotypeDX, n			0.309
Low risk (< 11)	124(21.1%)	154(18.3%)	
Medium risk (11–25)	357(60.7%)	516(61.3%)	
High risk (> 25)	106(18%)	171(20.3%)	
Not available	1(0.2%)	0(0%)	
HER2 RT-PCR median [IQR]	9.1[8.6–9.5]	9.2[8.8–9.7]	** < 0.00001**
HER2 RT-PCR < 8.5	116(19.7%)	93(11%)	
HER2 RT-PCR ≥ 8.5	472(80.3%)	748(89%)	** < 0.00001**
ER IHC, n			**0.0114**
0	0(0%)	0(0%)	
1	14(2.4%)	14(1.7%)	
2	70(11.9%)	64(7.6%)	
3	497(84.5%)	758(90.1%)	
Not available	7(1.2%)	5(0.6%)	
PR IHC, n			0.648
0	121(20.6%)	179(21.3%)	
1	64(10.9%)	110(13.1%)	
2	103(17.5%)	152(18.1%)	
3	281(47.8%)	387(46%)	
Not available	19(3.2%)	13(1.5%)	

The ER/PR score reflects the staining intensity of specimens graded as 0, 1, 2, and 3 as none, mild, moderate, and strong, respectively

*IDC* Invasive ductal carcinoma, *ILC* Invasive lobular carcinoma, *ER* Estrogen receptor, *PR* Progesterone receptor

^*^p value calculated with the t-test/Mann–Whitney test for continuous variables and the chi-square test for categorical variables. Bold indicates significance. The RT-PCR cut was determined by logistic regression (= 8.5)

**Table 2:** Contingency table analysis to evaluate the association between clinical pathological characteristics or adjuvant treatments and having a HER2-low result higher than the first

Parameter	Increased HER2 in the recurrence (*n* = 21)	No increase in HER2 in the recurrence (*n* = 23)	p^*^
Time to relapse (months), median [IQR]	50[39–76]	63[40.5–97]	0.25^**^
Time disease interval, n(%)			0.2
≤ 5 years	14(66.7%)	11(47.8%)	
> 5 years	7(33.3%)	12(52.2%)	
Patient received RT after the first RS, n (%)			1
Yes	21(100%)	22(95.7%)	
No	0(0%)	1(4.3%)	
Patient received ET after the first RS, n (%)			1
Yes	21(100%)	22(95.7%)	
No	0(0%)	1(4.3%)	
Duration of ET (months), median (IQR)	60(45–62.5)	60(39–66)	0.97
Patient received CT after the first RS, n (%)			0.22
Yes	11(52.5%)	8(34.8%)	
No	9(42.4%)	14(60.9%)	
Not available	1(4.8%)	1(4.3%)	
Timing of CT			
Adjuvant	20(95.2%)	18(78.3%)	
Neoadjuvant	1(4.8%)	3(13%)	
Not available	0(0%)	2(8.7%)	
Median (IQR) age at testing, years	57(50–66)	59(52–64)	0.76
Median (IQR) tumor size, cm	2.1(1.5–2.5)	2(1.7–2.95)	0.81
Tumor size category, cm, n (%)			
≤ 1 cm	1(4.8%)	1(4.3%)	
> 1–2 cm	9(42.7%)	12(52.2%)	0.87
> 2 cm	10(47.7%)	9(39.2%)	
Not available	1(4.8%)	1(4.3%)	
Tumor grade category, n (%)			0.28
Grade 1	0(0%)	2(8.7%)	
Grade 2	10(47.7%)	13(56.5%)	
Grade 3	8(38.1%)	5(21.7%)	
Not available	3(14.2%)	3(13.1%)	
Histological type, n (%)			0.86
IDC	13(61.8%)	17(74%)	
ILC	6(28.6%)	5(21.7%)	
Mucinous/colloid/papillary	1(4.8%)	1(4.3%)	
Other/not available	1(4.8%)	0(0%)	
Nodal status, n (%)			0.46
N0	10(47.7%)	8(34.8%)	
N1mi	4(19%)	5(21.7%)	
N1	4(19%)	8(34.8%)	
Not available/other	3(14.3%)	2(8.7%)	
OncotypeDX, median [IQR]	23[16.5–33]	24[15.5–28]	0.97
OncotypeDX, n (%)			0.75
Low (< 11)	3(14.3%)	2(8.7%)	
Medium (11–25)	8(38.1%)	12(52.2%)	
High (> 25)	9(47.6%)	9(39.1%)	
HER2 RT- PCR median [IQR]	9.25[8.75–9.5]	9.2[8.7–9.8]	0.43
HER2 RT- PCR < 8.5	3(14.3%)	2(8.7%)	0.63
HER2 RT- PCR = > 8.5	11(52.4%)	15(65.2%)	
Not available	7(33.3%)	6(26.1%)	
ER IHC, n (%)			0.49
0	0(0%)	0(0%)	
1	0(0%)	2(8.7%)	
2	0(0%)	1(4.3%)	
3	21(100%)	20(87%)	
PR IHC, n (%)			0.29
0	5(23.7%)	7(30.4%)	
1	1(4.8%)	3(13%)	
2	1(4.8%)	4(17.4%)	
3	14(66.7%)	9(39.2%)	
Surgery type			0.22
Lumpectomy	15(71.4%)	10(43.5%)	
Mastectomy	6(28.6%)	9(39.1%)	
Not available	0(0%)	4(17.4%)	
Site of metastasis, n (%)			
Bones			
Yes	12(57.1%)	15(65.2%)	0.58
No	9(42.9%)	8(34.8%)	
Liver			
Yes	8(38.1%)	9(39.2%)	0.94
No	13(61.9%)	14(60.8%)	
Lungs			0.86
Yes	5(23.8%)	6(26%)	
No	16(76.2%)	17(74%)	
Brain			0.17
Yes	4(19%)	1(4.3%)	
No	17(81%)	22(95.7%)	

*IDC* Invasive duct carcinoma, *ILC* Invasive lobular carcinoma, *ER* Estrogen receptor, *PR* Progesterone receptor, *RT* Radiotherapy, *CT* Chemotherapy, *ET* Endocrine therapy, *RS* Recurrence score

^*^p value calculated with the t test/Mann-Witney for continuous variables and the chi-square/Fisher test for categorical variables. Bold indicates significance

^**^one-tailed

Tumors were considered HR + or HER2-positive according to the latest American Society of Clinical Oncology guidelines [9]. HER2-negative tumors were further classified into 2 groups: HER2-0 for an IHC score of 0 and HER2-low for an IHC score of 1 + ‏ or 2 + ‏ with non-amplified FISH [10]. The majority of HER2 status assessments in this study were conducted via microscopy until 2021, with the exception of seven more recent metastatic samples that were evaluated digitally. The ER and PR scoring system (0, 1 + , 2 + , and 3 +) evaluates receptor expression based on the staining intensity and proportion of positively stained cells. Zero, 1 + , 2 + and 3 + reflect no expression, weak expression, moderate expression, and strong expression, respectively [11].

### Statistical analysis

To investigate differences between primary HER2 status (low & 0) and between paired relapse samples (increase in HER2 expression/no increase), Chi-square or Fisher’s exact tests were used for categorical variables, and a 2-tailed (unless stated otherwise) Student's t-test/Wilcoxon rank test was used for continuous variables. Univariate logistic regression was applied to estimate the relationship between HER2 IHC status (negative/low, as the dependent variable) and HER2 RT-PCR (as the independent predictor). We visualized the HER2 expression evolution from primary to metastatic BC by means of Sankey diagrams. A p value < 0.05 was considered significant for all analyses. Data calculation, statistical analysis, and graphics were carried out using R for Windows (https://www.r-project.org/, version 4.0.3 [12].

## Results

### Patient and tumor characteristics

We studied 1429 HR + /HER2-negative (IHC = 0/1 +, or IHC = 2 + and FISH not amplified) BC patients who underwent the OncotypeDX test (Table 1). Of 1429 patients, data on distant metastatic recurrence were available for 1226 patients, of whom 87 (7.1%) relapsed (distant metastases). Data regarding metastatic BC patients are presented in Tables 2 and 3. The median follow-up duration was 76.8 months. Forty-five patients underwent biopsy for distant metastases. The proportions of HER2-low and HER2-0 cases in the primary tumor cohort were 58.8% and 41.2%, respectively. The median follow-up duration time was 80.5 months, and the median time to relapse was 55 months for the 87 patients in the metastatic cohort.

**Table 3:** HER2 expression evolution from primary to relapsed breast cancer

		**Metastatic BC HER2 classification n(%)**			**Total**
		HER2-0	HER2-Low	HER2-Positive	
**Primary BC HER2 classification, n(%)**	HER2-0	6(13.6)	9(20.5)	2(4.5)	17(38.6)
	HER2-Low	9(20.5)	13(29.5)	5(11.4)	27(61.4)
**Total**		15(34.1)	22(0.5)	7(15.9)	44(100)

### Differences between HER2-0 and HER2-low in the OncotypeDX-tested population

**Fig. 1:**
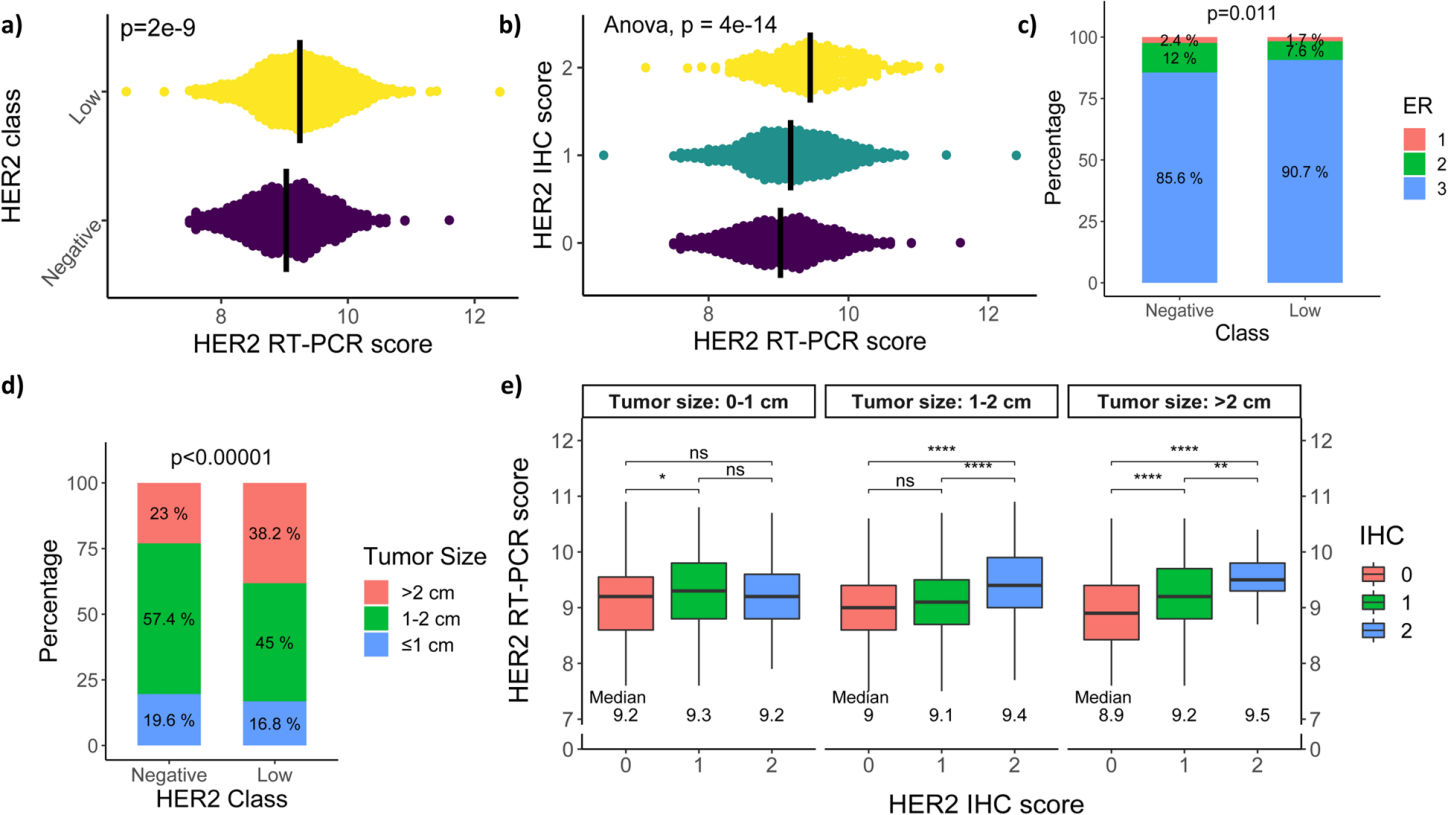
Differences between HER2-0 and HER2-low in the OncotypeDX population. **a**,**b** HER2 RT-PCR significantly differentiates not only HER2-low from HER2-0 BC (*p* < 0.0001) but also between HER2 IHC = 0/1 + /2 + categories (*p* < 0.0001). The black line indicates the group mean. The density indicates the number of overlapping samples. **c** HER2-low cases in primary tumors expressed higher ER levels (*p* = 0.0114) and were larger **d** than HER2-0 cases (*p* < 0.00001) **e** Differences between HER2 RT-PCR and HER2 IHC results, stratified by tumor size groups. HER2 RT-PCR significantly differentiates between HER2 IHC scores, especially in > 2 cm tumors, but it does not contribute to the distinction in tumors < 1 cm. Medians are specified under each group

HER2-low cases in primary tumors had a higher HER2 RT-PCR score (*p* = 2.1e-9; Fig. 1 a and b), expressed higher ER IHC levels (*p* = 0.0114; Fig. 1c), and were larger (> 2 cm; 36.6% versus 22.1%, *p* < 0.00001; Fig. 1 d) compared with the HER2-0 cases. Tumors > 2 cm were more likely to be HER2-low (OR = 2.07, 95% CI: 1.6317 to 2.6475, *p* < 0.0001). We further examined the correlation between HER2 RT-PCR and IHC results stratified by tumor size groups. As shown in Fig. 1e, for larger tumors, the median differences significantly increased in correlation with HER2 RT-PCR. No significant differences were observed for the other examined characteristics. HER2 RT-PCR differentiated not only HER2-low from HER2-0 BC but also between HER2 IHC = 0/1 + /2 + categories (Fig. 1b). The RT-PCR cut-off was determined by logistic regression (= 8.5). The mean values ± standard deviation of HER2 RT-PCR for HER2 IHC 0, 1 + , and 2 + were 9.01 ± 0.76, 9.17 ± 0.69, and 9.45 ± 0.66, respectively.

### Metastatic cohort characteristics and differences in HER2 expression between primary and metastatic biopsies

**Fig. 2:**
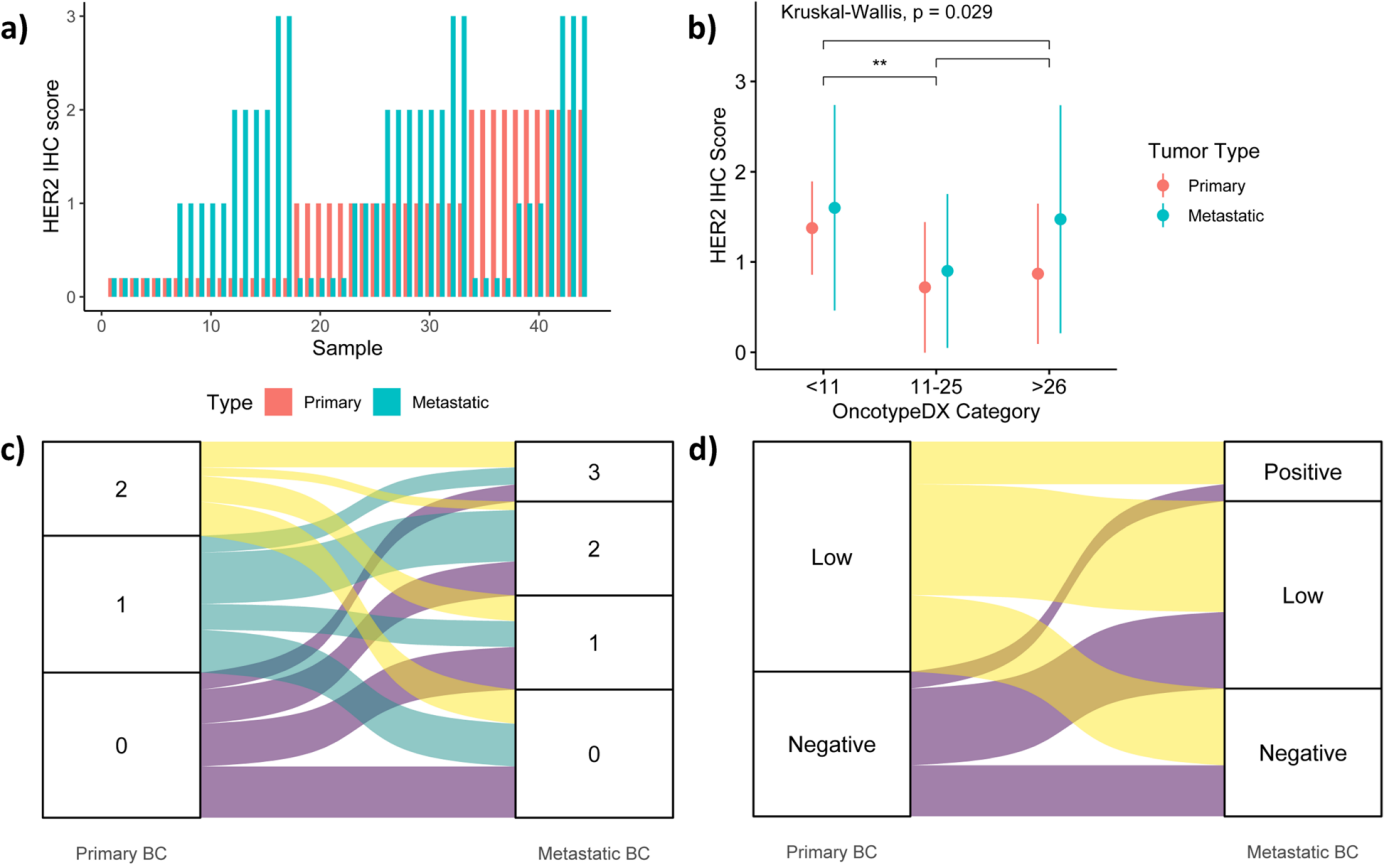
Metastatic characteristics and differences in HER2 expression between primary and metastatic biopsies.** a** HER2 IHC scores in primary vs. metastatic BC for each patient in the paired study cohort. Metastatic BCs had a significantly higher HER2 IHC score than primary BCs (one-tailed Wilcoxon signed-rank, *p* = 0.046).** b** Differences in HER2 IHC score by OncotypeDX category in primary and metastatic BCs, including all primary BC biopsies and unpaired metastatic BC biopsies. The low-risk OncotypeDX group had a higher IHC score than the medium-risk group (*p* = 0.0067). Dots represent means. **c, d** Transition of HER2 expression from primary to metastatic BC. Most of the patients gained or maintained some degree of HER2 expression (65.9%)

Metastatic BC had a significantly higher HER2 IHC score compared with primary BC (1-tailed Wilcoxon signed-rank, *p* = 0.046; Fig. 2a). Forty-four samples of metastatic recurrence were eligible for pairing. Common biopsy sites included the liver (15), bones (12), lungs (7) and skin [6]. We used a contingency table analysis to evaluate the association between clinical pathological characteristics or adjuvant treatments and having a metastatic HER2 IHC result higher than the initial score ("increase" vs. "no increase"). The increase group had a shorter time to relapse and more numerous brain metastases (19% vs. 4.3%, *p* = 0.17), but neither these nor other parameters reached a level of significance (Table 2).

The low-risk OncotypeDX group had a higher HER2 IHC score than the medium-risk group, as shown in an unpaired comparison between primary and metastatic BC samples comprising all the available biopsies, based upon the OncotypeDX score category (duns *p* = 0.0067; Fig. 2b). The majority of patients (65.9%) gained or maintained some degree of HER2 expression (Table 3). Figure 2 c and d summarize the HER2 evolution from primary BC to metastatic BC. The overall discordance rate was 56.8% (*n* = 25), largely comprising samples switching to or from HER2-low expression. Specifically, 52.9% of HER2-0 primary BCs switched to the HER2-low phenotype, and 11.8% of the HER2- primary BCs evolved to the HER2-positive phenotype, while 33% of the HER2-low primary BCs switched to the HER2-0 phenotype, and 18.5% of the HER2-low primary BCs became HER2-positive. Of note, out of the 12 metastatic samples originating from bone, 50% had the HER2-0 phenotype in comparison to 37.5% of samples that were biopsied elsewhere. Among the 44 metastatic samples, seven that were evaluated digitally did not demonstrate higher scores compared to microscopically assessed counterparts (Mann–Whitney, *p* = 0.92).

## Discussion

We characterized several factors that differentiate the HER2-low group from the HER2-0 group. Our results showed a positive correlation between a higher RT-PCR score and a HER2-low status, suggesting that RT-PCR could potentially enhance the prediction capability of the traditional IHC test in borderline cases. HER2-low patients are expected to express higher levels of mRNA compared with HER2-0 patients, and their results should therefore be more commonly found at the higher end of the RT-PCR scale. This partially explains the overlap between IHC subgroups (Fig. 1 a and b) and enables us to focus upon the area of the scale where there is a high percentage of patients classified as HER2-low. We further showed that the correlation between HER2 RT-PCR and IHC results, stratified by tumor size groups, was enhanced for larger tumors (Fig. 1e). These results reinforce the notion that HER2 RT-PCR can be a good indicator of HER2 IHC status, especially in tumors larger than 2 cm, but it does not contribute to the distinction in tumors smaller than 1 cm. Indeed, prior studies have yielded analogous findings, indicating a positive association between higher RT-PCR scores and HER2-low status. However, those studies were constrained by much smaller cohort sizes [13, 14].

We also found that larger tumors were significantly associated with HER2-low status. Given that HER2 belongs to the epidermal growth factor receptor family, whose activation results in cell proliferation and tumorigenesis [15], it is not surprising that tumors that overexpress HER2 will be characterized by accelerated proliferation and growth in size compared with tumors that do not overexpress HER2. A comparison of ER IHC levels with HER2-0 and -low status also linked higher ER IHC levels to HER2-low status. This finding raises the possibility of crosstalk between HER2 and HR pathways and may contribute to HER2-low phenotype variation, as observed in other studies [5, 16].

We showed a significant increase in HER2 expression compared with primary BC in distant metastases (Fig. 2), and that finding correlates with tumors being more aggressive, as reported by others [5, 17]. A comparison of the HER2 evolution between primary and matched distant-staged BCs revealed that the majority of patients (65.9%) gained or maintained some degree of HER2 expression. HER2 pathway expression has also been suggested as a mechanism of resistance to hormonal treatment, thereby explaining HER2 gain (even at low levels) in metastatic samples [18]. Interestingly, we showed that based upon the OncotypeDX score category, the low-risk group (< 11) had a higher HER2 IHC score than the medium-risk group (11–25). Despite not reaching a level of significance, brain metastases were more common among patients with increased HER2 vs. no-increase HER2 patients. Several studies have demonstrated that patients with HER2-positive BC are more commonly affected by brain metastases than HER2-negative/HR + patients [19]. This correlation may also be consistent with HER2-low BC compared with HER2-0 BC. Further research with a larger paired cohort could elucidate the mechanism underlying this finding.

The HER2 treatment paradigm changed with the emergence of TDXd as the only FDA-approved medication to treat HER2-low patients. Drug eligibility lies entirely upon IHC test findings, and incorrect classification may result in missing patients who qualify for its use. Technical limitations (staining techniques), interobserver differences, intratumoral heterogeneity, or insufficient sensitivity can undermine the reliability of IHC as the sole test for classification. Indeed, the HER2-enriched subtype was infrequent and similarly distributed in HER2-low and HER2-0 BCs [20]. HER2 intratumoral heterogeneity has been linked to low-grade or HER2 IHC = 2 + BCs in several reports [21–24]. Another study showed that HER2 intratumoral heterogeneity was also more frequent in HER2-low cases than in HER2-positive cases, and those authors emphasized that smaller tumor samples are more prone to inaccurate assessment of HER2 status [23]. Moreover, primary tumors are larger than metastatic biopsies and therefore are less susceptible to false assessment.

Intratumoral heterogeneity of HER2 has an effect in another direction as well. Internalization of TDXd releases a payload of DXd in the target cell in addition to having a bystander effect, which causes death of neighboring cells regardless of their HER2 status [7]. Bearing intratumoral heterogeneity in mind, even patients classified by IHC as HER2-0 might potentially benefit from the drug. Tissue heterogeneity, technical limitations and bystander effects point to whether it is appropriate to insist upon taking a sample from a metastatic tumor such as in DESTINY-Breast04 [7], which is smaller, or rely on sample results from the larger primary tumor. ESMO guidelines emphasize new biopsies upon metastatic relapse [25]. However, when infeasible, treatment aligns with primary tumor traits. ASCO also allows oncologists to rely on primary samples, given that metastatic samples are more susceptible to pre-analytic conditions than primary breast tissue samples [26]. The decision-making process should also evaluate the prognostic costs of false positive and false negative classifications in terms of a patient who will needlessly receive the medication or a suitable patient who will be ruled out.

Artificial intelligence (AI) offers a promising solution for the inaccuracies of IHC/FISH testing, enhancing precision and inter- and intraobserver reproducibility while potentially reducing the need for molecular testing [27, 28]. Nevertheless, Wu et al. (2023) highlight that AI's impact on accuracy was most pronounced among novice pathologists [27]. Similarly, Palm et al. (2023) suggest AI's potential in distinguishing positive and negative HER2 samples, which encountered challenges in HER2 IHC 0/1 + classification [28]. A potential implication of our observation could be by implementation of the OncotypeDX with HER2 levels by AI to overcome pathologists' subjectivity. AI thus represents a significant advancement, hinting that a multidisciplinary approach could potentially provide the most accurate solution.

This study has several limitations. First, we used only paired samples to characterize the evolution of HER2. It is possible that we would have obtained more significant results had the paired cohort been larger. In addition, this is a single-center study with no access to follow-ups from other institutions. Although it may create some degree of bias, the rates of metastatic recurrence and the long median follow-up duration are similar to those described in other studies. Furthermore, our results are consistent with those of other reported analyses in terms of the median follow-up time [29] and HER2 enrichment [5, 17]. Another limitation stems from the majority of metastatic tumor biopsies having been collected during/after ET, which potentially altered the HER2 expression result. However, ET duration was practically identical among the HER2 increase vs. no-increase HER2 groups. Indeed, digital evaluation of images can sometimes lead to higher estimates compared to microscopic images. Nevertheless, in our research, the number of digital samples was limited, and no statistical difference was observed.

Evaluation of HER2 status in bone metastases via IHC is susceptible to underestimating HER2 scores due to the decalcification process [30]. Therefore, prioritizing nonbone sites for biopsies is favorable. Indeed, we observed that 50% of bone samples were classified by IHC as 0. Nonetheless, considering that bone is the most likely site for breast metastases to reach, we believe it is advisable not to overlook data originating from bone altogether.

Differences among antibodies can affect HER2 assessment. Ventana's 4B5 (which was predominantly used in our institution) is less sensitive than DAKO HercepTest (polyclonal and new monoclonal) [31]. Rüschoff et al. (2022) [32] found that DAKO HercepTest (new monoclonal) was more specific than Ventana's 4B5 and DAKO HercepTest (polyclonal). We claim that HER2 IHC = 0/1 + by Ventana might still benefit from TDXd because Ventana's 4B5 might underestimate the true extent of HER2-low tumors.

The new HER2-low classification and the implications of using ADCs in this setting have revolutionized breast oncology. A debate surrounds whether HER2-low status affects BC biology, necessitating gene expression analysis, or if the receptor's presence alone suffices as a therapeutic target for ADC binding, irrespective of pathway activation. In SABCS 2022, both perspectives are presented [33]. Should the latter perspective hold true, it would call into question that patients with HER2-0 status by IHC paired with pathway activation through HER2 RT-PCR would gain benefits from therapies such as TDXd. Additionally, clinical evidence for ADCs beyond traditional IHC remains limited.

Taken together, our study findings demonstrate that HER2-low status is associated with a higher RT-PCR score, larger tumor size, and higher ER expression. We also showed enrichment of HER2 in the metastatic setting. Revisiting primary BC settings could complement the imperfect widely applied IHC test, which would be especially relevant to borderline cases. These findings may not only enable us to more accurately characterize HER2-low but also provide HER2-low patients with more appropriate treatment, potentially expanding the patient population eligible for novel anti-HER2 treatment.

## Data Availability

The datasets used and/or analyzed during the current study are available from the corresponding author upon reasonable request.

## Abbreviationss

ET: Endocrine therapy
CT: Chemotherapy
BC: Breast cancer
IHC: Immunohistochemistry
HER2: Human epidermal growth factor receptor
HR: Hormone receptor
ER: Estrogen receptor
PR: Progesterone receptor
TDXd: Trastuzumab deruxtecan
RS: Recurrence score
ADCs: Antibody-drug conjugates
